# Impact of unintended initial dissection of the posterior plane during SMILE surgery on surgery time and visual outcomes

**DOI:** 10.1186/s12886-022-02333-x

**Published:** 2022-03-08

**Authors:** Ke Zheng, Yinan Han, Jing Wang, Tian Han, Xingtao Zhou

**Affiliations:** 1 Ophthalmology Department, Eye & ENT Hospital, Fudan University, Shanghai, China; 2 NHC Key Laboratory of Myopia (Fudan University), Shanghai, China; 3 Key Laboratory of Myopia, Chinese Academy of Medical Sciences, Shanghai, China; 4 Shanghai Research Center of Ophthalmology and Optometry, Shanghai, China

**Keywords:** Lenticule, Small incision lenticule extraction, Complication, Unintended dissection, Cap lenticular adhesion

## Abstract

**Background:**

To study the impact of unintended initial dissection of the posterior plane (UIDPP) on operation time and surgical outcomes during small incision lenticule extraction (SMILE) surgery.

**Methods:**

This was a retrospective study. Based on the SMILE procedure video, the operating eyes were assigned to the normal and UIDPP groups according to the presence or absence of UIDPP signals during surgery. The UIDPP group was further separated into early and late detection based on whether the complete dissection of the lenticule posterior plane or not. Patient's demographic data, preoperative evaluation data, operation time and postoperative outcomes were collected.

**Results:**

Sixty-six patients (66 eyes) who underwent SMILE were included, with 24 eyes with UIDPP (13 in the early detection group and 11 in the late group). The optical zone was smaller (median 6.5 vs. 6.6, *P* = 0.007), and the operation time was longer (median, 189.5 vs. 91.0 s, *P* < 0.001) in the UIDPP group compared with normal group. There were significant differences in operation time between the late detection group and early detection group (median, 489.0 vs. 139.0 s, *P* < 0.05) and between the late detection group and normal group (median 489.0 vs. 91.0 s, *P* < 0.05), while the optical zone was different only between the late detection and normal groups (median, 6.5 vs. 6.6, *P* < 0.05). At the one-year follow-up, UDVA was better than or equal to 20/20 in 87.5% of eyes, and 75% of eyes were within ± 0.5 D of the intended refractive target. One eye lost one Snellen line.

**Conclusion:**

The occurrence of UIDPP will significantly prolong the operation time, but not affect the recovery of long-term visual acuity after surgery. Detecting UIDPP earlier could help shorten the operation time.

## Introduction

Myopia is a common refractive problem in which the images focus in front of the retina, leading to distant objects appearing blurry [1]. Myopia affects about 1.5 billion people, or 22% of the world population [1, 2], reaching 37% in large Chinese cities [1, 3]. Astigmatism is another type of refractive error in which the images do not focus evenly on the retina due to differences in refractive power for light coming from different directions [4], resulting in blurry vision at any distance [4, 5]. In Europe and Asia, astigmatism of different levels affects 30%-60% of individuals [5]. The management of both eye conditions includes wearing glasses or contact lenses and refractive surgery [2, 5].

Refractive surgery is used in patients who desire independence from glasses and contact lenses. Various methods are available, but the most commonly used is excimer lasers. Recent years have seen the widespread application of femtosecond laser in ophthalmology. The available technologies include laser-assisted in situ keratomileuses (LASIK) and small incision lenticule extraction (SMILE) [6, 7]. SMILE has demonstrated good safety, effectiveness, stability, and predictability [8, 9].

Still, as a surgical procedure, SMILE carries surgical risks and specific complications [10]. According to the expert consensus on SMILE in China and elsewhere [11, 12], after laser scanning, the dissection of the anterior surface (under the corneal cap) of lenticule is recommended, followed by the posterior surface (posterior plane) of the lenticule. Especially, corneal transparency makes this dissection process challenging. Less experienced doctors are likely to accidentally dissect the posterior plane in advance in the initial stage, but the anterior plane of the lenticule has not yet been dissected. Because the lenticule adheres to the corneal cap [13], it is then challenging to continue to dissect the lenticule for less experienced doctors, which is called the unintended initial dissection of the posterior plane (UIDPP) [14, 15]. Still, the clinical impacts of UIDPP are poorly understood.

In this setting, the present study explored whether UIDPP affected the procedure time and postoperative effect and whether the early detection of intraoperative complications and late detection had a different impact. The results could provide a basis for the clinical evaluation and treatment of UIDPP during SMILE.

## Methods

### Study design and patients

The retrospective study included myopia patients who underwent SMILE at the Eye & ENT Hospital of Fudan University from July 2015 to September 2017. During this period, the patients underwent bilateral SMILE sequentially. The patients with intraoperative complications were excluded, including intraoperative corneal cap margin tear or corneal epithelial damage at the incision, suction loss, lenticule tear, lenticule off-centering, opaque bubbles, dark areas in the corneal stroma scanning area, difficulty in identifying simple posterior plane, and residual lenticule or unsuccessful removal. This study followed the tenets of the Declaration of Helsinki and was approved by the Ethics Committee of the Eye and ENT Hospital of Fudan University. Informed consent was obtained from all participants.

Based on the procedure video, the operating eyes were assigned to the normal and UIDPP groups according to the presence or absence of UIDPP signals during surgery. The UIDPP group was classified based on the complete dissection of the lenticule posterior plane: the early detection group (UIDPP was detected before the complete dissection of the posterior plane) and the late detection group (UIDPP was detected after the complete dissection of the posterior plane).

### Standard surgical procedure

All procedures were performed by the same surgeon(KZ). The surgeon in the present study has finished more than 100 LASIK, LASEK and PRK.

The general process and the conditions need to be cooperated with in the surgery have been informed patient in detail before the operation. After topical anesthesia of the operating eyes, the VisuMax femtosecond laser (Carl Zeiss GmbH, Oberkochen, Germany) was used to prepare a lenticule. The frequency was set to 500 kHz. The thickness of the corneal cap was 120 µm. The diameter of the corneal cap was 7.5 mm. The cap margin incision was at 12 o'clock, and the size was 2 mm. After femtosecond laser cutting, a dissector was used for dissecting lenticule, and microsurgical forceps were used to remove the lenticule. During the dissection, the surgeon used the left hand to control the eye with forceps and used the right hand to dissect the lenticule with a spatula (model No.52435 T; 66 Vision-tech Corp., Suzhou, China).

### Data collection

Patients’ demographic data, preoperative evaluation data (slit-lamp microscopy, mydriasis fundoscopy, snellen eye chart for uncorrected distance visual acuity (UDVA), diopter of sphere and cylinder, and manifest refraction spherical equivalent (MRSE) and best-corrected visual acuity (BCVA), postoperative uncorrected visual acuity (UCVA), refractive state the optical zone, cutting depth, operation time were collected. The differences in the duration of intraoperative dissection and removal of lenticule were analyzed. The dissector contacting the corneal cap incision was used as the starting time. The lenticule being completely removed from the corneal cap was the ending time. The time difference between the two-time points was the operation time. UDVA, MRSE, and BCVA of patients before surgery and more than one year after surgery and UCVA one month after surgery were collected.

### Statistical analysis

All eligible patients during the study period were included. Microsoft Excel for MAC version 15.24 was used to analyze dissection time. Data were analyzed using SPSS 26.0 (IBM, Armonk, NY, USA). The Kolmogorov–Smirnov test was used to test the normality of data in each group. Non-normally distributed continuous data were presented as median (interquartile range) and analyzed using the non-parametric Mann–Whitney U-test (two groups) and the non-parametric Kruskal–Wallis test (more than two groups) with the least-significant difference post hoc test. Categorical data were presented as n (%) and analyzed using the chi-square test. *P* < 0.05 was considered statistically significant.

## Results

### Characteristics of the patients

In the present study, 66 patients (66 eyes) underwent SMILE during the study period. There were 30 males (45.4%) and 36 females (54.5%). The age range was 17–37 years, with a mean of 23.6 ± 4.6 years. There were 24 eyes in the UIDPP group, including 13 in the early detection group (13 eyes) and 11 in the late detection group (11 eyes). All operating eyes were successfully dissected, and the lenticule was removed. The procedure time of lenticule dissection and removal of all patients ranged from 47 to 908 s.

Table 1 describes the characteristics of the patients in the normal and UIDPP groups. There were no statistically significant differences between the two groups in age, sex, MRSE, preoperative BCVA, scotopic pupil, intraocular pressure, central corneal thickness, axial length, and white-to-white distance (all *P* > 0.05). There were no statistically significant differences between the early and late detection groups in age, sex, MRSE, preoperative BCVA, scotopic pupil, intraocular pressure, central corneal thickness, axial length, and white-to-white distance (all *P* > 0.05).

**Table 1:** Characteristics of the patients

Characteristic	Normal group (*n* = 42)	UIDPP group (*n* = 24)		P (normal vs. UIDPP)	P (early vs. late)
		UIDPP early detection (*n* = 13)	UIDPP late detection (*n* = 11)		
Age, years (median (IQR))	24 (7.5)	22 (8)	21 (5)	0.246	0.495
Sex (male), n (%)	20 (47.6)	4 (30.8)	6 (54.5)	0.582	0.441
Spherical equivalent, median (IQR)	-4.5 (2.50)	-5.5 (2.06)	-6.00 (3.75)	0.403	0.700
BCVA, median (IQR)	1.2 (0)	1.2 (0)	1.2 (0.2)	0.256	0.437
Scotopic pupil, median (IQR)	7.2 (1.1)	7.0 (0.8)	7.0 (1.1)	0.298	0.562
Intraocular pressure, mmHg (median (IQR))	16.2 (4.1)	14.8 (2.85)	16.2 (2.9)	0.329	0.472
Corneal thickness, µm (median (IQR))	537 (40.5)	527 (62)	531 (49)	0.467	0.761
Axial length, mm (median (IQR))	25.87 (1.50)	25.48 (1.37)	25.85 (1.59)	0.292	0.567
White to white distance, mm (median (IQR))	11.9 (0.4)	12 (0.25)	12 (0.4)	0.956	0.997

*UIDPP* Unintended initial dissection of the posterior plane, *IQR* Interquartile range, *BCVA* Best-corrected visual acuity

### Postoperative outcomes

The differences in cutting depth and UCVA one month after surgery are not statistically significant with *P* > 0.05, while the optical zone was smaller (median 6.5 vs. 6.6, *P* = 0.007) and the operation time was longer (median, 189.5 vs. 91 s, *P* < 0.001) in the UIDPP group compared with normal group (Table 2). Among the normal, early detection and late detection groups, the optical zone and operation time were statistically different, *p* = 0.025 and < 0.001 respectively. Comparing the three groups in pairs, the difference of operatin time between late and early detection group (median, 489.0 vs. 139.0 s, *P* < 0.05) and late detection and normal group (median 489 vs. 91 s, *P* < 0.05) were both significant. While the optical zone was different only between the late detection and normal groups (median, 6.5 vs. 6.6, *P* < 0.05) (Table 3).

**Table 2:** Comparison of the postoperative parameters between the normal group and UIDPP group

	Operation time (second)			Optical zone (mm)			Cutting depth (um)			UCVA 1 month after surgery		
	min	max	median (IQR)	min	max	median (IQR)	min	max	median (IQR)	min	max	median (IQR)
Normal	47	235	91.0 (90.5)	6	6.7	6.6 (0.2)	58	152	104.0 (37.5)	1	1.2	1.2 (0)
UIDPP	90	908	189.5 (326)	6	6.7	6.5 (0.08)	53	142	113.5 (39.75)	0.8	1.5	1.2 (0.2)
P	< 0.001			0.007			0.510			0.925		

*UIDPP* Unintended initial dissection of the posterior plane, *IQR* Interquartile range, UCVA Uncorrected visual acuity

**Table 3:** Comparison of postoperative parameters among the early detection group, late detection group, and normal group

	Operation time(second)			Optical zone(mm)			Cutting depth(um)			UCVA 1 month after surgery		
	min	max	median (IQR)	min	max	median (IQR)	min	max	median (IQR)	min	max	median (IQR)
Normal	47	235	91.0 (90.5)	6	6.7	6.6 (0.2)	58	152	104.0 (37.5)	1	1.2	1.2 (0)
Early detection	90	284	139.0 (82)	6.1	6.7	6.5 (0.05)	63	142	114.0 (35.5)	1	1.5	1.2 (0.5)
Late detection	102	908	489.0 (486) *#	6	6.7	6.5 (0.3) *	53	142	103.0 (46)	0.8	1.5	1.0 (0.2)
P	< 0.001			0.025			0.783			0.345		

^*^ *P* < 0.05 vs. the normal group; ^#^ *P* < 0.05 vs. the early detection group

*IQR* Interquartile range, *UCVA* Uncorrected visual acuity

**Fig. 1:**
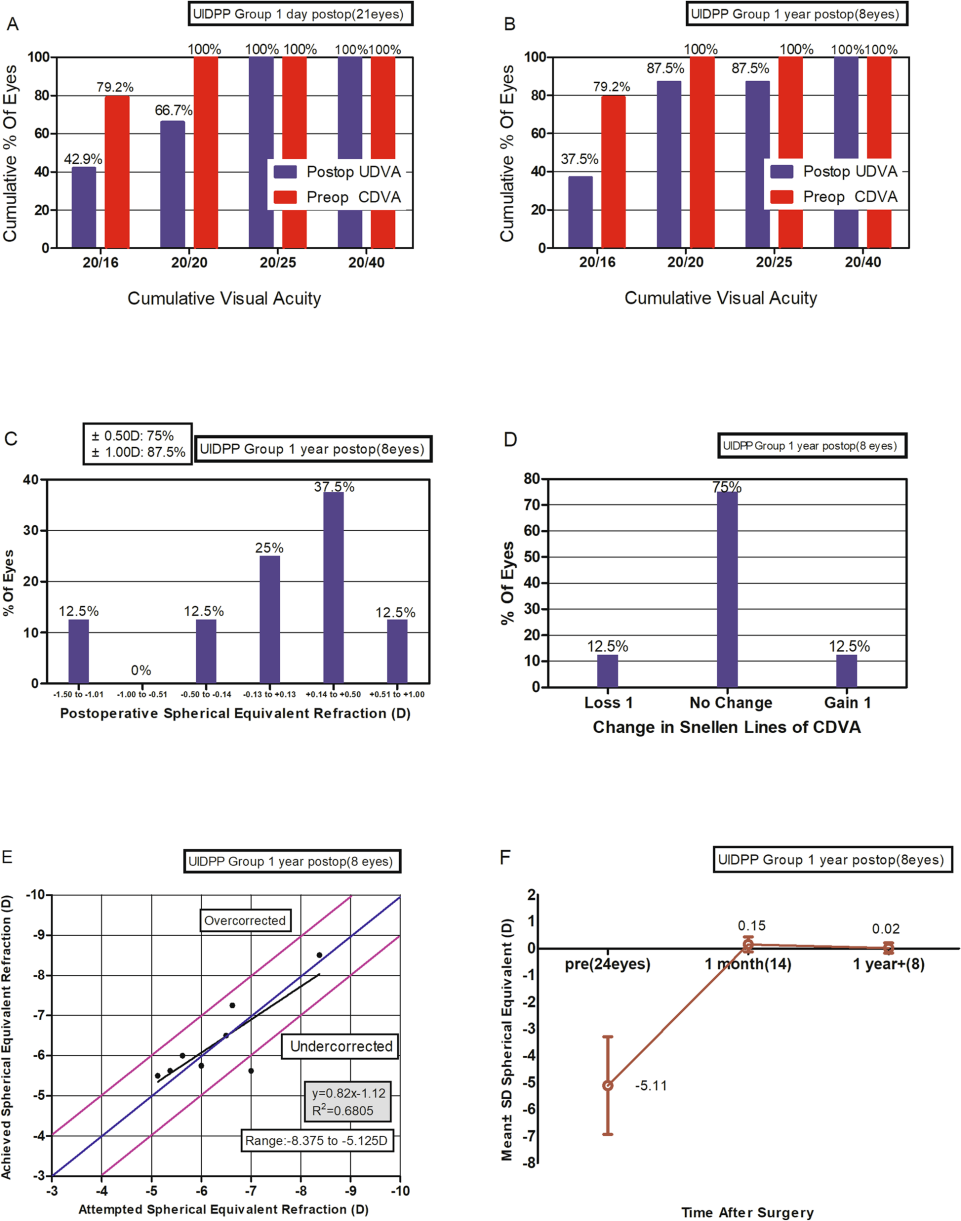
Follow-up results of the UIDPP group before surgery and 1 day and over 1 year after surgery. **A** Comparison of uncorrected visual acuity on the first day after surgery and preoperative corrected visual acuity. **B** Comparison of uncorrected visual acuity 1 year after surgery and preoperative corrected visual acuity. **C** Equivalent spherical distribution 1 year after surgery. **D** Changes in best-corrected visual acuity 1 year after surgery. **E** Predictive distribution 1 year after surgery. **F** Stability of refraction 1 year after surgery

Figure 1 shows the follow-up results of the UIDPP group before surgery, one day, and one year after surgery. At the one-year follow-up, UDVA was better than or equal to 20/20 in 87.5% of eyes(Fig. 1B), and 75% of eyes were within ± 0.5 D of the intended refractive target(Fig. 1E). One eye lost one Snellen line(Fig. 1D).

## Discussion

This study aimed to study the effect of UIDPP on operation time and surgical outcomes during SMILE surgery. The results indicate that the occurrence of UIDPP will increase the surgery time of SMILE. Detecting UIDPP earlier could help shorten the operation time.

There are many contributors to UIDPP during SMILE, including 1) abnormal laser energy, dark areas, and opaque bubble layer that cause the lenticule to adhere to the corneal cap, 2) abnormal corneal tissue structure, such as a narrow distance between the corneal cap margin and the lenticule edge incision, can cause difficult dissection at the inferior corneal cap (upper surface of the lenticule) or the lower surface of the lenticule, 3) excessive eye movement, 4) due to the transparency of the lenticule, it is difficult for the surgeon to distinguish the upper and lower surfaces of the lenticule, and it is easy to dissect the posterior plane, and 5) the lenticule anterior plane tightly adheres to the surface of the anterior stroma, increasing the incidence of UIDPP during surgery [16]. Ivarsen et al. [16] proposed that the preoperative parameters of eyes with difficult lenticule dissection were not different from those of normal ones, but Shetty et al. [13] conducted an analysis of 550 operating eyes undergoing SMILE and found that the operating eyes with low MRSE, low corrected spherical diopter, and thin lenticule more easily had UIDPP during surgery, causing adhesion of corneal cap and lenticule.

There are subjective and objective factors in the causes of UIDPP. The surgeon's lack of experience or the thinness of the lenticule can lead to initial dissection of the posterior plane. Even experienced surgeons can experience UIDPP [13, 17], not only novices [18].

In the present study, the analysis of 66 eyes showed that UIDPP might be associated with the optical zone, but not with sex, age, spherical equivalent, BCVA, scotopic pupil, intraocular pressure, corneal thickness, axial length, and white-to-white distance. It could be used as a reference for novices to design the size of the optical zone for SMILE.

The present study showed that the mean MRSE and UCVA of patients with UIDPP tended to be stable. Studies by Qiu & Yang [19] showed that intraoperative complications could cause potential vision loss, while Wang et al. [17] found that the postoperative UCVA of patients with UIDPP during surgery reached 1.0 or more through the analysis of SMILE procedures and six months of follow-up. The average spherical diopter was -0.25 D, and the eyesight was good. Ramirez-Miranda et al. [20] believed that most complications during SMILE were associated with the surgeon's experience, not affecting the patient's final vision. Shetty et al. [13] suggested that longer procedure time might cause postoperative slight visual distortion without affecting central visual acuity; of the ten eyes with initial dissection of the posterior plane, five had slight interlayer opacity on the first day after surgery, and all returned to normal three months after surgery; eight eyes showed mild myopia (< 3 D), and the other two showed moderate myopia (3–6 D). Zheng et al. [21] reported that difficult plane dissection in SMILE was associated with left eyes for right-handed physicians, in eyes with low spherical equivalent, of eyes with high J0 values. In the present study, UIDPP generally does not affect the one-year follow-up vision after surgery, but excessive procedures might have a certain effect on the corneal stroma. Effects of difficult lenticule dissection caused by UIDPP on the long-time effect remain to be determined.

The difficult lenticule dissection and removal caused by UIDPP during SMILE are the most important conditions for refractive procedure surgeons, especially novices, who need to avoid [18]. For the procedure time of SMILE, the time for lenticule dissection and removal is important. In the present study, the operation time of the UIDPP group was longer than that of the normal group. Furthermore, the operation time was longer for the late detection group than for the early detection group. The reason might be that after complete dissection of the posterior plane, if the surgeon does not realize that initial dissection of the posterior plane has occurred and continues to find the posterior plane, then the operation time will be very long. Even if the surgeon has already realized that the initial dissection of the posterior plane has occurred because the lenticule tightly adheres to the corneal cap, it is difficult to quickly find the anterior plane to complete the dissection when the dissector searches upward. The present study found that if surgeons realized the dissector had entered the posterior plane before completing the dissection of the posterior plane and stopped continuing the dissection of the posterior plane in time, at this time the lenticule did not completely adhere to the corneal cap, it would be easier to find the anterior plane, and the operation time would be decreased. Therefore, the results suggested that the early discovery of UIDPP is crucial for less experienced surgeons, a series of possible intraoperative prompt signals [21–23] for early detection of the lenticule should be notied.

This study has limitations. The sample size was small and from a single hospital. Only one surgeon was involved. The retrospective nature of the study limited the analyzable data to those that were in the charts. Additional studies are necessary to compare the dissection time after unintended initial dissection of the posterior plane between novice and experienced surgeons.

## Conclusion

UIDPP during the SMILE procedure greatly increases the procedure time. Detecting UIDPP before the complete dissection of the posterior plane could avoid the prolonged procedure time. However, UIDPP had no effect on long-term visual recovery. Further research is warranted regarding the longer-term effect of UIDPP on postoperative visual outcomes.

## Data Availability

All the data used to support the findings of this study are included within the article and are available from the corresponding author upon reasonable request.

## Abbreviations

UIDPP: Unintended initial dissection of the posterior plane
SMILE: Small incision lenticule extraction
UDVA: Uncorrected distance visual acuity
MRSE: Manifest refraction spherical equivalent
BCVA: Best-corrected visual acuity
UCVA: Uncorrected visual acuity
